# The register shift rules for *βαβ*-motifs for *de novo* protein design

**DOI:** 10.1371/journal.pone.0256895

**Published:** 2021-08-30

**Authors:** Hiroto Murata, Hayao Imakawa, Nobuyasu Koga, George Chikenji

**Affiliations:** 1 Department of Applied Physics, Graduate School of Engineering, Nagoya University, Nagoya, Aichi, Japan; 2 Protein Design Group, Exploratory Research Center on Life and Living Systems (ExCELLS), National Institutes of Natural Sciences (NINS), Okazaki, Aichi, Japan; 3 SOKENDAI, The Graduate University for Advanced Studies, Shonan Village, Hayama, Kanagawa, Japan; 4 Research Center of Integrative Molecular Systems, Institute for Molecular Science (IMS), National Institutes of Natural Sciences (NINS), Okazaki, Aichi, Japan; Chuo University, JAPAN

## Abstract

A wide range of *de novo* design of *αβ*-proteins has been achieved based on the design rules, which describe secondary structure lengths and loop torsion patterns favorable for design target topologies. This paper proposes design rules for register shifts in *βαβ*-motifs, which have not been reported previously, but are necessary for determining a target structure of *de novo* design of *αβ*-proteins. By analyzing naturally occurring protein structures in a database, we found preferences for register shifts in *βαβ*-motifs, and derived the following empirical rules: (1) register shifts must not be negative regardless of torsion types for a constituent loop in *βαβ*-motifs; (2) preferred register shifts strongly depend on the loop torsion types. To explain these empirical rules by physical interactions, we conducted physics-based simulations for systems mimicking a *βαβ*-motif that contains the most frequently observed loop type in the database. We performed an exhaustive conformational sampling of the loop region, imposing the exclusion volume and hydrogen bond satisfaction condition. The distributions of register shifts obtained from the simulations agreed well with those of the database analysis, indicating that the empirical rules are a consequence of physical interactions, rather than an evolutionary sampling bias. Our proposed design rules will serve as a guide to making appropriate target structures for the *de novo* design of *αβ*-proteins.

## Introduction

*De novo* protein design allows us to explore the whole protein sequence space in principle and create proteins with brand new structures and functions, independently from any naturally existing proteins [1]. Recently, significant progress has been made in *de novo* protein design and many successful examples have been reported, such as proteins with new shape [2–10] and new therapeutic proteins [11–14]. One of the procedures used for *de novo* design of proteins with *β*-sheets consists of the following three steps: (1) Determinig a blueprint of the target structure, a two-dimensional map specifying the number and lengths of secondary structures and loop torsion angle bins represented by the ABEGO classification [15], etc. (2) Building a three-dimensional backbone structure based on the blueprint. (3) Searching for amino acid sequences that fold to the target structure. In this procedure, the determining blueprints is important because if blueprints specify the three-dimensional structures that are physically undesignable, the *de novo* design invariably fails. In fact, some studies demonstrated that inappropriate blueprints for *β*-sheet-containing structures resulted in the failure of the *de novo* design [7, 10]. Then, how can we identify blueprints that specify designable three-dimensional structures?

Blueprints for *de novo* protein design have been created based on the rules for physically preferred local backbone geometries [1]. Examples of the rules include, the length of the loop controls the packing orientation of *ββ*-, *βα*-, and *αβ*-units [3], side chain directionality determines the preferred loop types connecting unpaired *β*-strands of *β*-sandwich structures [8], and large local deviations in the ideal *β*-strand twist are necessary to form a closed *β*-barrel [7]. Incorporating these rules into a blueprint made it possible to design proteins with shapes that could not have been designed rationally before [1]. Therefore, discovering new rules will contribute to the development of *de novo* protein design technology. For designing proteins containing *β*-sheets, the following parameters are required for making blueprints, secondary structure lengths [4]; loop geometries [8]; locations of bulges [6]; register shifts [5], etc. Here, a register shift is identified as a residue offset between terminal residues of adjacent *β*-strands. Among these parameters, the design rules specifying register shifts have not been reported, even though they are necessary. To create a blueprint that leads to successful design of *αβ*-proteins, understanding the design rules of register shifts from a physical viewpoint is essential. Here, we propose the rules for register shifts in *βαβ*-motifs, and provide the physical origin by physics-based simulation.

## Results and discussion

### Definition of register shift for *βαβ*-motifs

**Fig 1:**
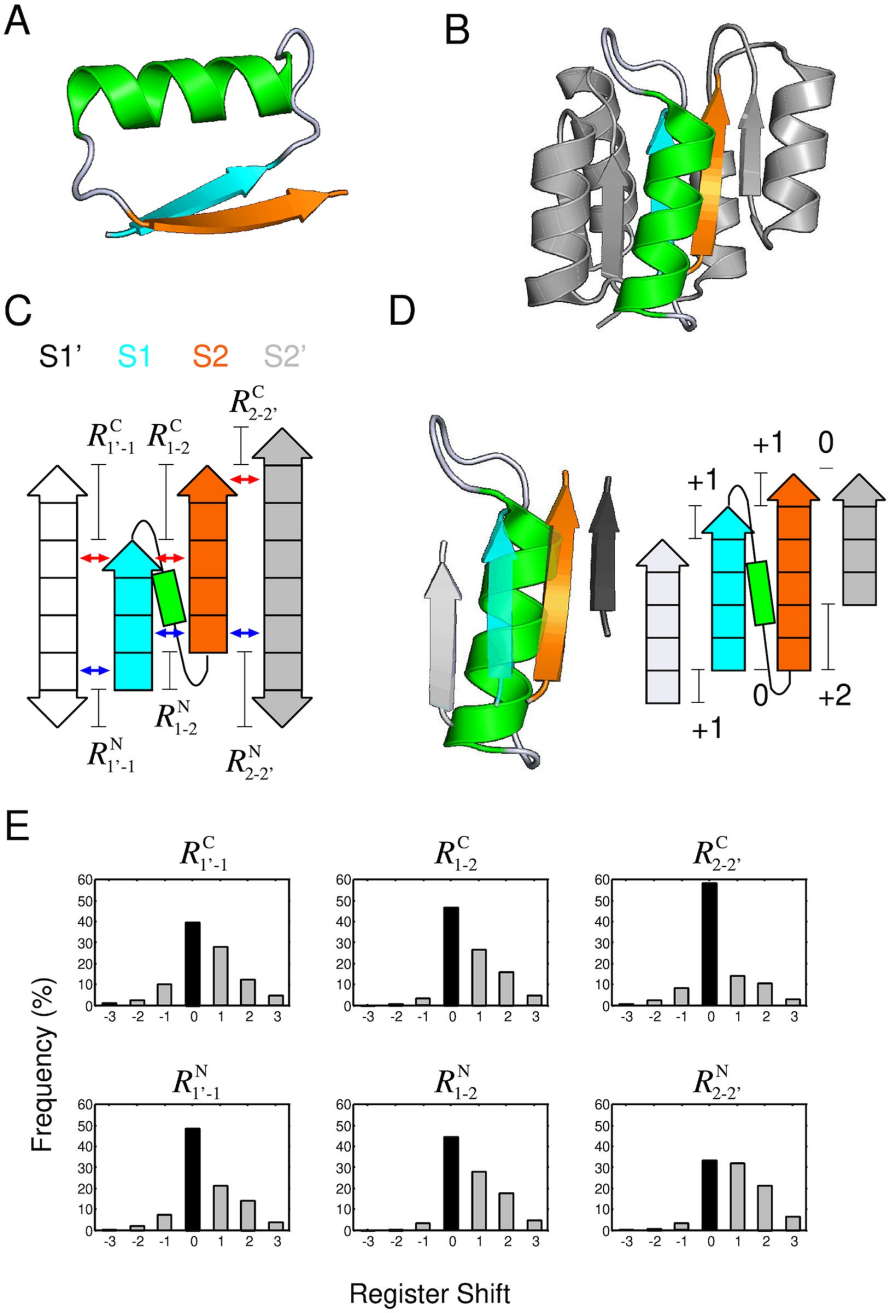
The six-type register shifts for *βαβ*-motifs, and the preferences in naturally occurring protein structures. (A) A *βαβ*-motif structure. A *βαβ*-motif is composed of two *β*-strands joined by an *α*-helix and forms a packing structure of the *α*-helix on the paired strands. The first and second strands are respectively colored in cyan and orange, and the *α*-helix is colored in green. (B) A naturally occurring protein structure containing *βαβ*-motifs (the response regulator Spo0F, PDB ID: 1peyA). In the structure, one of the *βαβ*-motifs is shown in colors. (C) A graphical explanation of the register shifts using a schematic representation of a *βαβ*-motif consisting of two *β*-strands (S1 and S2) and an *α*-helix, with two neighboring *β*-strands, S1’ and S2’. To make it clear that the direction of S1’ and S2’ can be either direction in the present database analysis, they are depicted by two-headed arrows. The blue and red arrows indicate the most N- and C-terminal residue pairs that form a cross-strand residue pairing, respectively. (D) The zoom-up view of the *βαβ*-motif, colored in (B), with the neighboring strands (Left), and its register shifts(Right). (E) Observed frequencies for each resister shift, $R_{1^{\prime }-1}^{C}$, $R_{1-2}^{C}$, $R_{2-2^{\prime }}^{C}$, $R_{1^{\prime }-1}^{N}$, $R_{1-2}^{N}$ and $R_{2-2^{\prime }}^{N}$, in naturally occurring protein structures. The bars at the register shift of zero, corresponding to the peak for the distributions, are indicated by filled black bars.

This section introduces *βαβ*-motifs and defines its register shifts. A *βαβ*-motif consists of two parallel *β*-strands belonging to the same *β*-sheet and an *α*-helix connecting the two strands with a right-handed connection (Fig 1A and 1B) [16]. Fig 1C shows a schematic representation of a *βαβ*-motif with two neighboring *β*-strands. In the figure, the N-terminal *β*-strand of the *βαβ*-motif (S1), the C-terminal *β*-strand (S2), and the *α*-helix connecting the two *β*-strands are colored in cyan, orange, and green, respectively. The neighboring *β*-strand paired with S1 is referred to as S1’ (white), and the one paired with S2 is referred to as S2’ (gray). S1’ and S2’ are depicted by two-headed arrows, to make it clear that we included S1’ and S2’ regardless of their (N- to C-terminus) chain directions in the present database analysis. The most N- and C-terminal residue pairs that form a cross-strand residue pairing are indicated by blue and red arrows, respectively. The definition of the cross-strand residue pairing is given in Ref. [17] and graphical explanation is shown in S1 Fig.

The register shifts for the *βαβ*-motif are defined as follows. Let *i*^*N*^(*X*) and *i*^*N*^(*X*, *Y*) be the residue number of the N-terminal residue of the X *β*-strand and the most N-terminal residue among residues of the X *β*-strand forming a cross-strand residue pairing with a residue of the Y *β*-strand, respectively (see S1 Fig). Using these notations, the N-terminal register shift for a parallel *β*-strand pair of S1 and S2 (denoted by $R_{1-2}^{N}$) is defined as

$$\begin{array}{c} R_{1-2}^{N}=|i^{N}\left(\text{S1},\;\text{S2}\right)-i^{N}\left(\text{S1}\right)|-|i^{N}\left(\text{S2},\;\text{S1}\right)-i^{N}\left(\text{S2}\right)|. \end{array}$$

Similarly, the C-terminal register shift of a pair of S1 and S2 (denoted by $R_{1-2}^{C}$) is defined as

$$\begin{array}{ccc} R_{1-2}^{C} & = & |i^{C}\left(\text{S2},\;\text{S1}\right)-i^{C}\left(\text{S2}\right)|-|i^{C}\left(\text{S1},\;\text{S2}\right)-i^{C}\left(\text{S1}\right)|, \end{array}$$

where *i*^*C*^(*X*) and *i*^*C*^(*X*, *Y*) are the residue number of the C-terminal residue of the X *β*-strand and the most C-terminal residue among residues of the X *β*-strand forming a cross-strand residue pairing with the Y *β*-strand, respectively.

When performing a database analysis of the register shifts of *βαβ*-motifs, we only included conformations with one or more *β*-strands on both sides of a *βαβ*-motif, as shown in Fig 1C. Here, any chain connectivity and either (N- to C-terminus) chain direction of S1’ and S2’ were considered. The purpose of including S1’ and S2’ is to avoid artifacts regarding the possible values of register shifts caused by the presence of S1 or S2 at the edge of the *β*-sheet. For example, if S1 is at the edge of the *β*-sheet, structures with a register shift of $R_{1-2}^{N}=+1$ or $R_{1-2}^{C}=-1$, as shown in S2A Fig, are not possible in principle because there is no hydrogen bonding partner for the residues at the terminal of S1 and, thus, they cannot exist as a *β*-strand residue. Conversely, if S1 is not at the edge of the *β*-sheet, a structure with a register shift of $R_{1-2}^{N}=+1$ or $R_{1-2}^{C}=-1$ could, in principle, exist (see S2B Fig). The same argument can be made about S2 (see S2C and S2D Fig). Therefore, depending on whether S1 and S2 are at the edge of the *β*-sheet, the possible register shift values can be restricted. To eliminate such artifacts, in the database analysis of register shifts, we focused exclusively on conformations with one or more *β*-strands on both sides of *βαβ*-motifs.

As shown later, the distribution of register shifts for the strand pair S1 and S1’ and that S2 and S2’ also exhibited an interesting behavior. The N-terminal and C-terminal register shifts between S1 and S1’ are expressed as $R_{1^{\prime }-1}^{N}$ and $R_{1^{\prime }-1}^{C}$. Similarly, those between S2 and S2’ are expressed as $R_{2-2^{\prime }}^{N}$ and $R_{2-2^{\prime }}^{C}$. The graphical explanation of these register shifts is shown in Fig 1C, and the mathematical definition is provided in Materials and methods section. Using these expressions, the register shifts of the *βαβ*-motif depicted in Fig 1D is given as $\left(R_{1^{\prime }-1}^{C},R_{1-2}^{C},R_{2-2^{\prime }}^{C},R_{1^{\prime }-1}^{N},R_{1-2}^{N},R_{2-2^{\prime }}^{N}\right)=\left(+1,+1,0,+1,0,+2\right)$.

### Database analysis of register shifts

We performed a statistical analysis of the six register shifts ($R_{1^{\prime }-1}^{C}$, $R_{1-2}^{C}$, $R_{2-2^{\prime }}^{C}$, $R_{1^{\prime }-1}^{N}$, $R_{1-2}^{N}$ and $R_{2-2^{\prime }}^{N}$) for a subset of protein structures deposited in the protein structure databank (PDB). The culled PDB dataset generated by the PISCES server [18] was used with the following parameters: resolution, ≤ 2.5Å; R-factor, ≤ 1.0; and sequence identity, ≤ 25%. For this dataset, we used the STRIDE program [19] for secondary structure assignment, from which *βαβ*-motifs with *β*-strands on both sides were identified. The number of identified *βαβ*-motifs was 5,776. For each motif, we identified the six register shifts and obtained their respective distributions.

The observed frequencies of the six register shifts in the dataset are shown in Fig 1E. The striking features of these graphs were as follows. (1) Common to all six graphs, a register shift of zero was the most frequently observed, indicating that the start or end points of spatially adjacent *β*-strands tend to be aligned. (2) For all six types of distributions, the occurrence of negative shifts was rare compared with the positive one. In particular, $R_{1-2}^{C}$, $R_{1-2}^{N}$, and $R_{2-2^{\prime }}^{N}$ exhibited few negative shifts. Later in this paper, we show that the negative register shifts of $R_{1-2}^{N}$ were physically prohibited for the most frequently observed loop type in *αβ*-units.

**Fig 2:**
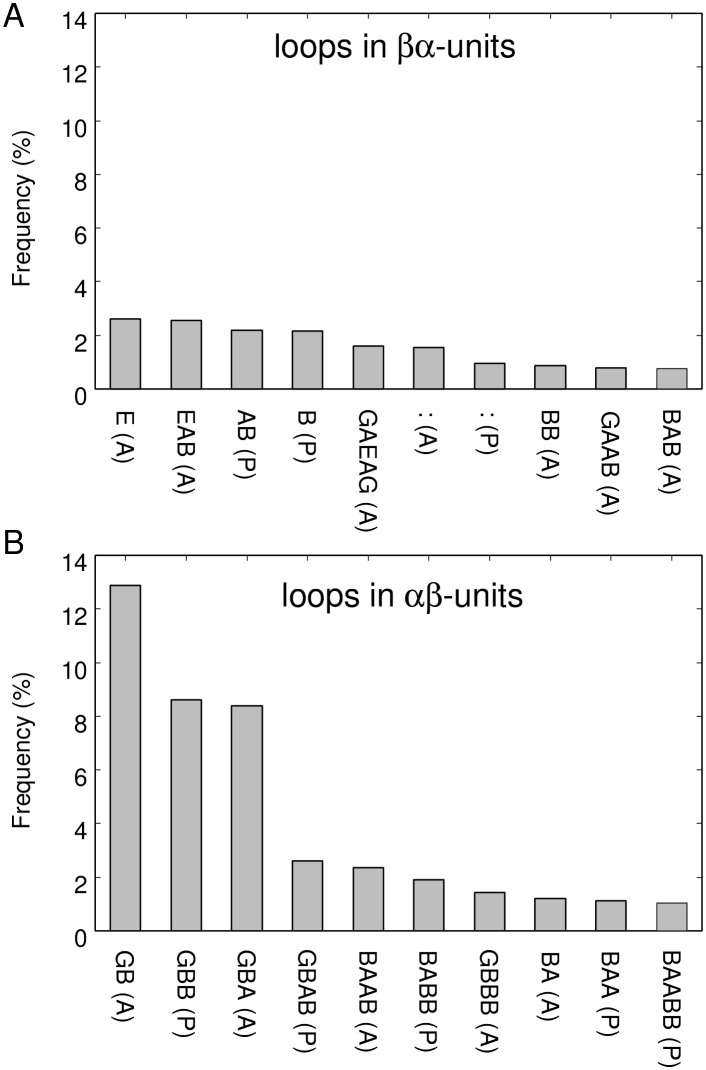
Observed frequency of loop types in *βα* (A) and *αβ* (B) units, as classified based on the ABEGO representation and packing geometry. The horizontal axis is sorted by frequency of occurrence and the 10 most frequently observed loops are shown. The packing geometry, i.e., parallel or anti-parallel, was defined based on the orientation of the C_*α*_-C_*β*_ vector of the strand residue closest to the helix relative to the vector from the first to the second secondary structure element [4]. If the two vectors were parallel, the orientation was denoted as “(P)”, and if anti-parallel, the orientation was denoted as “(A)”. The character “:” denotes the zero-length loop.

As reported in the literature [4], loops in *αβ*-units and those in *βα*-units exhibit various torsion types. It would be instructive to investigate whether the trend of the statistical distribution of register shifts depends on the loop types included in *βαβ*-motifs. Before conducting this analysis, we performed a census of loop types in our dataset to obtain an overview of the degree of variation in loop types. Fig 2 shows the occurrence frequencies for loop types in *βα*-units and those in *αβ*-units, as classified according to the ABEGO representation [4, 15, 20] and the packing geometry (parallel or anti-parallel) [4]. Here, the 10 loops with the highest frequency of occurrence are shown. In the ABEGO classification, “A”, “B”, “E”, “G”, and “O” denote the right-handed *α*-helix region of the Ramachandran plot; right-handed *β*-strand region; left-handed *β*-strand region; left-handed helix region; and the cis peptide conformation, respectively. As shown in Fig 2(A) and 2(B), there was no predominant loop type in loops in *βα*-units, whereas there were three prominently more frequent loop types in loops in *αβ*-units; i.e., GB(A), GBB(P), and GBA(A). This result suggests that loops in *βα*-units are highly diverse, whereas those in *αβ*-units exhibits less diversity. In fact, in this dataset, there were 2,749 loop types in *βα*-units and only 910 loop types in *αβ*-units, indicating that loop types in *αβ*-units have a limited variation compared with those in *βα*-units. In the following, we refer to GB(A) loop in *αβ*-units as GB loop because GB(P) loop was not observed in the dataset, erasing the need to distinguish between GB(A) and GB(P). Similarly, we will refer to GBA(A) loop in *αβ*-units as GBA loop.

Do distributions of register shifts depend on the loop types of components of a *βαβ*-motif? The observed register shifts for each loop type in *βα* and *αβ*-units listed in Fig 2 are shown in S3–S6 Figs These figures indicates that the distributions of register shifts vary significantly according to the loop types and that the distributions for some loop types (e.g., $R_{2-2^{\prime }}^{N}\text{(GB)}$) largely differed from the average distribution of all loop types shown in Fig 1E.

**Fig 3:**
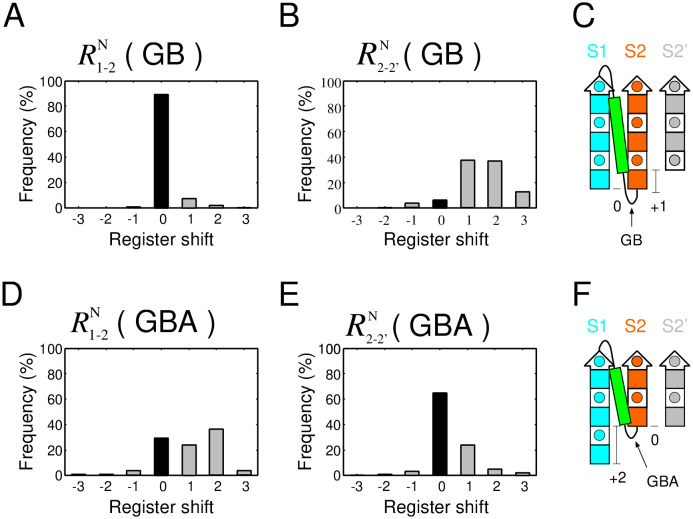
The distribution of register shifts of the two most frequently observed loop types with anti-parallel orientation in *αβ*-units in the dataset. (A) Distribution of the register shift of $R_{1-2}^{N}\text{(GB)}$, and (B) that of $R_{2-2^{\prime }}^{N}\text{(GB)}$. (C) Schematic structure of a *βαβ*-motif with the most frequent register shift of $R_{1-2}^{N}\text{(GB)}$ and $R_{2-2^{\prime }}^{N}\text{(GB)}$. (D) Distribution of the register shift of $R_{1-2}^{N}\text{(GBA)}$, and (E) that of $R_{2-2^{\prime }}^{N}\text{(GBA)}$. (F) Schematic structure of a *βαβ*-motif with the most frequent register shift of $R_{1-2}^{N}\text{(GBA)}$ and $R_{2-2^{\prime }}^{N}\text{(GBA)}$. In (C) and (F), the square with a circle inside represents a single amino acid residue with side chain located on the proximal side, and the colored filled square represents a residue with a side chain located on the opposite side.

As interesting examples, the distributions of $R_{1-2}^{N}\text{(GB)}$ and $R_{1-2}^{N}\text{(GBA)}$, the register shifts of the two most frequent loops with anti-parallel orientation in *αβ*-units, are shown in Fig 3. In the $R_{1-2}^{N}\text{(GB)}$ distribution, almost only $R_{1-2}^{N}\text{(GB)}=0$ was observed (Fig 3A), whereas in the $R_{1-2}^{N}\text{(GBA)}$ distribution, $R_{1-2}^{N}\text{(GBA)}=0,1,2$ was observed with roughly the same frequency (Fig 3D). In addition, for $R_{2-2^{\prime }}^{N}\text{(GB)}$, a register shift of zero was rarely observed (Fig 3B), whereas for $R_{2-2^{\prime }}^{N}\text{(GBA)}$, the observed frequency of a register shift of zero was prominently large (Fig 3E). Therefore, the difference between the distribution of register shifts of GB and GBA loop was large, implying that the preferred register shifts strongly depend on loop type.

### Method for exhaustive structural sampling of model peptides and physical interaction calculations

Can the distributions of register shifts shown in Fig 3, S3 and S4 Figs be attributed to an evolutionary sampling bias, or are they an inevitable consequence of physical interactions? In the following, we show that the distribution of $R_{1-2}^{N}\text{(GB)}$ and $R_{2-2^{\prime }}^{N}\text{(GB)}$, as shown in Fig 3, can be explained by physical interactions, as demonstrated by an exhaustive structural sampling of model peptides and physical interaction calculations.

The outline of this calculation is as follows. First, we generated exhaustive GB loop structures with an *α*-helix on their N-termini and a *β*-strand connected to their C-termini, and obtained various *αβ*-unit structures. Then, we placed each of them within a *β*-sheet consisting of three *β*-strands with various register shifts, and identified the structures that met the various conditions. The conditions imposed here were the exclusion volume [21] and the hydrogen bond satisfaction conditions [22]. The exclusion volume condition based on the hard-sphere model stipulates that the distance between any two atoms cannot be less than the sum of their van der Waals radii. The hydrogen bond satisfaction condition implies that the polar backbone atoms must form hydrogen bonds, either intramolecularly or with a solvent. Fitzkee *et al*. showed that the conformational constraints imposed by these two rules are sufficient to reproduce the fragment conformations observed in PDB [23], suggesting that breaking either of the two rules is such a strong violation that it is forbidden in native structures. We show that structural ensembles satisfying the two conditions were qualitatively consistent with the distributions obtained from the database analysis reported in Fig 3A and 3B.

We used a 12-residue peptide as a model for a *αβ*-unit connected by a GB loop (see S7 Fig). For this system, we selected an amino acid sequence made up solely of alanine residues, except for the ninth residue, which was glycine, because this position is the G position in the GB loop; thus, the dihedral angle at this position is the G region of the ABEGO classification; therefore, glycine is most physically preferred. In fact, only glycine was prominently abundant at the G position in the GB loop in the dataset (see S8 Fig). The structure of the peptide from 1st to the 8th residue was constrained to be an *α*-helix, and that of the 12th residue to be a *β*-strand. The internal coordinates of these residues (dihedral angle, bond angle, and bond length) were fixed to the values of those of the consensus structure of the *αβ*-unit connected by a GB loop. The consensus structure was defined as the one with the highest similarity to all structures of GB loops in *αβ*-units in the dataset. More specifically, given *N* *αβ*-units connected by a GB loop, the sum of the root-mean-square deviations (RMSDs) to all other structures from a structure *i*,

$$\begin{array}{c} S\left(i\right)=\sum_{j\neq i}^{N}\text{RMSD}\left(i,j\right), \end{array}$$ (1)

was calculated and the structure with the smallest *S*(*i*) was selected as the consensus structure. The consensus structure was the structure of residues 85–96 of a putative oxidoreductase (PDB ID: 3c1aB). For this model peptide, *ϕ* and *ψ* angles of the 9th, 10th, and 11th residues were exhaustively sampled within the G, B, and B regions shown in S9 Fig, respectively, by subdividing the *ϕ*-*ψ* space into a 5° × 5° grid. Since, there were 145 and 277 discrete states in the region G and B, respectively, the total number of exhaustively sampled structures was 11,125,705 (= 145 × 277 × 277). Among the generated *αβ*-unit structures, only conformations preserving the hydrogen bonds of the consensus structure and met the the steric exclusion condition were used in the next stage of the experiment. Here, for evaluating the steric exclusion condition, only the heavy atoms of the main chain and the C_*β*_ atoms were considered, and the hard sphere atomic radii described in Ref. [21] were used. For identifying hydrogen bonds, the HBPLUS program [24] was used. The number of structures that met the two conditions was 1,586,681.

**Fig 4:**
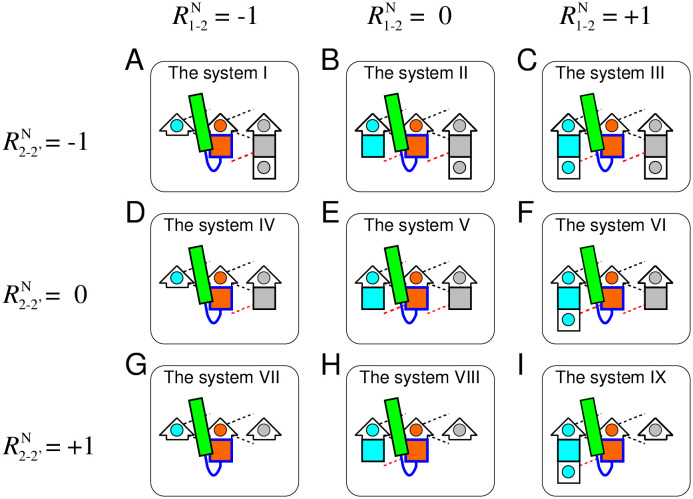
Schematic representations of nine target systems for all atom simulation. Figures (A)–(I) show schematic representations of the system I–IX, respectively. Each system consists of the *αβ*-unit and the three-stranded *β*-sheet structure with different register shifts. The arrows represent *β*-strands and the rectangles represent *α* helices. In the *β*-strands, residues located at the C-terminus are indicated by short arrows, and other residues are shown as squares. A square (or a short arrow) with a circle inside represents a single amino acid residue with a side chain that is on the proximal side, and a colored filled square represents a residue with a side chain on the opposite side. The register shifts $\left(R_{1-2}^{N},R_{2-2^{\prime }}^{N}\right)$ of the systems I to IX were (−1, −1), (0, −1), (1, −1), (−1, 0), (0, 0), (1, 0), (−1, 1), (0, 1), and (1, 1), respectively. The residues indicated by blue are residues of which *ϕ* and *ψ* angles were exhaustively sampled. The black dotted lines indicate hydrogen bonds that were fixed in the exhaustive sampling, while the red dotted lines indicate hydrogen bonds that can be broken.

Each generated *αβ*-unit structure that satisfied the two abovementioned conditions was implanted in three-stranded *β*-sheet structures with the nine different register shifts shown in Fig 4. The systems shown in Fig 4A–4I are termed as the system I–IX, respectively. The three-dimensional structures of the three-stranded *β*-sheet were obtained from the consensus structure of the three-stranded *β*-sheets with lengths of five. The reason for the choice of the five-residue *β*-strand is documented in Materials and methods section. The method used for determining the consensus structure was essentially the same as that for determining the consensus structure of the *αβ*-unit, except the use of the MICAN algorithm [25–27], a method that ignores the connectivity of the *β*-strands for computing RMSDs. The consensus structure obtained was the structure of residues 5–9, 36–40, and 106–110 of the bacterial cell division regulator protein MipZ (PDB ID: 2xj4A). Only the heavy atoms of the main chain and the C_*β*_ atoms were used for the evaluation (these atoms are shown in S10 Fig). The *β*-sheet structures with the nine different register shifts shown in Fig 4 were prepared by removing the atoms from the consensus *β*-sheet structure. All atoms of *β*-sheet structures of each of the system I–IX are shown in S11–S19 Figs. Into each of the nine *β*-sheet structures, each exhaustively generated conformations of the *αβ*-unit was implanted. The procedure for this implantation is documented in Materials and methods section.

For a given conformation of each implanted system, we evaluated whether the conformation satisfied the following three conditions.

(a) **The *β*-sheet hydrogen bond condition**: The hydrogen bonds necessary for each *β*-sheets shown in Fig 4 must be correctly formed. The list of the hydrogen bond conditions required for each *β*-sheet system is shown in Table 1. The intra-chain hydrogen bonds used for evaluating *β*-sheet formation are indicated by red dotted lines in Fig 4. Definition of the hydrogen bonds listed in Table 1 is provided in S20 Fig. Note that the system VII cannot satisfy this condition at all because there is no hydrogen bonding partner for the N-terminal residue of S2 and thus it cannot, in principle, be a *β*-strand residue.
(b) **The steric exclusion condition**: Atomic collisions between the *αβ*-unit and S1 or S2’ are prohibited. The hard sphere atomic radii described in Ref. [21] were used for evaluating the steric exclusion condition.
(c) **The hydrogen bond satisfaction condition**: Any donor and acceptor in the main chain must form hydrogen bonds, either intramolecularly or with a water solvent. The CHASA program [28] was used to determine whether a polar group that did not form an intra-chain hydrogen bond can undergo hydrogen bonding with a water molecule.

We repeated this evaluation for all exhaustively generated structures in each system, and identified the number of structures that satisfy the three conditions for the system I–IX.

**Table 1:** List of intra-chain hydrogen bonds that must/must not be satisfied for the systems I–IX. The HB1 bond is defined as the hydrogen bond between the donor of the most N-terminal residue of the S1 strand in the system III, VI, or IX and the acceptor of the first residue of the GB loop. The HB2 and the HB3 bonds are defined as the hydrogen bonds required for the system of $R_{1-2}^{N}\geq 0$ and $R_{2-2^{\prime }}^{N}\leq 0$, respectively. The graphical explanations of HB1, HB2, and HB3 are given in S20 Fig. The symbol “b” means that it must be bonded, “-” means that its hydrogen bond is ignored because a given system does not include such a donor-acceptor pair, and “n” means that it must not be bonded. HB1 is “n” in the systems III, VI, and IX because if HB1 is formed, the N-terminus of S2 will be stretched by one residue; therefore, it will not be a required register shift for these systems.

hydrogen bond\system	I	II	III	IV	V	VI	VII	VIII	IX
HB1	-	-	n	-	-	n	-	-	n
HB2	-	b	b	-	b	b	-	b	b
HB3	b	b	b	b	b	b	-	-	-

### The computational results were consistent with the statistics obtained in the database analysis

**Fig 5:**
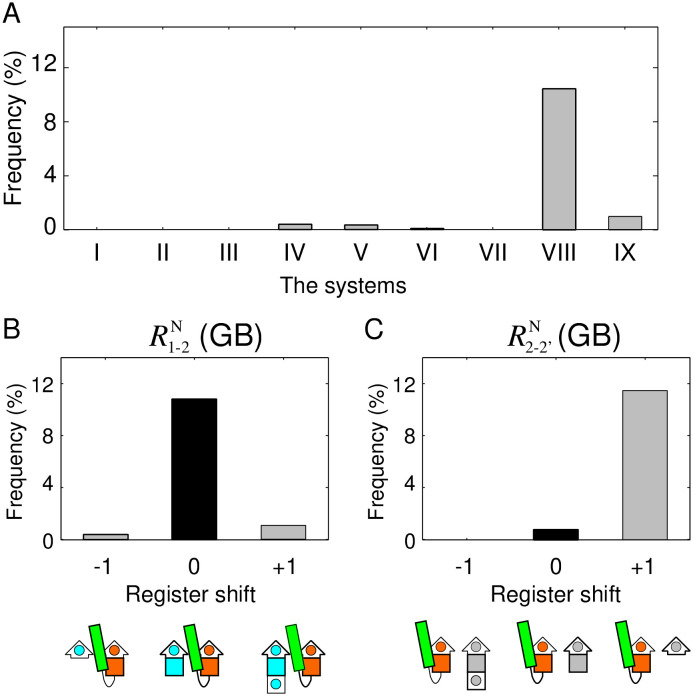
The results of exhaustive conformational sampling and identification of structures satisfying the conditions. (A) Frequency of structures that satisfy the three conditions among exhaustively generated structures for the system I to IX. (B) Distributions of the computed register shifts of $R_{1-2}^{N}\text{(GB)}$ and (C) that of $R_{2-2^{\prime }}^{N}\text{(GB)}$. The values of the vertical axis of (B) and (C) were obtained by marginalizing the results of (A).

Fig 5A presents the occurrence frequency of structures that satisfied the three conditions for the nine systems shown in Fig 4. It is evident from the figure that the occurrence frequency of structures that satisfied all the three conditions was prominently large only in the system VIII, and almost zero in the other systems. This result implies that the system VIII alone, which has the register shift of $\left(R_{1-2}^{N},R_{2-2^{\prime }}^{N}\right)=\left(0,1\right)$, is physically suitable for a GB loop, and that the other systems are almost physically prohibited.

Next, we demonstrate that the results of these calculations are consistent with the statistics of the database analysis shown in Fig 3A and 3B. To compare them, we computed the occurrence frequencies for register shifts of $R_{1-2}^{N}\text{(GB)}=-1$, $R_{1-2}^{N}\text{(GB)}=0$, $R_{1-2}^{N}\text{(GB)}=+1$, $R_{2-2^{\prime }}^{N}\text{(GB)}=-1$, $R_{2-2^{\prime }}^{N}\text{(GB)}=0$, and $R_{2-2^{\prime }}^{N}\text{(GB)}=+1$. Here, we assume that their frequencies denoted by *P* can be obtained by the following equations.

$$\begin{array}{ccc} P(R_{1-2}^{N}\text{(GB)}=-1) & = & f(\text{A})+f(\text{D})+f(\text{G})\\ P(R_{1-2}^{N}\text{(GB)}=\;\;0) & = & f(\text{B})+f(\text{E})+f(\text{H})\\ P(R_{1-2}^{N}\text{(GB)}=+1) & = & f(\text{C})+f(\text{F})+f(\text{I})\\ P(R_{2-2^{\prime }}^{N}\text{(GB)}=-1) & = & f(\text{A})+f(\text{B})+f(\text{C})\\ P(R_{2-2^{\prime }}^{N}\text{(GB)}=\;\;0) & = & f(\text{D})+f(\text{E})+f(\text{F})\\ P(R_{2-2^{\prime }}^{N}\text{(GB)}=+1) & = & f(\text{G})+f(\text{H})+f(\text{I}). \end{array}$$

Here, *f*(*x*) denotes the frequency of structures satisfying the conditions in the system *x* (from I to IX). The resulting distributions of $P(R_{1-2}^{N}\text{(GB)})$ and $P(R_{2-2^{\prime }}^{N}\text{(GB)})$ are shown in Fig 5B and 5C, respectively. The comparison of the register shift distribution shown in Fig 5B with the distribution of the range of the horizontal axis from −1 to 1 in Fig 3A showed that both are in good agreement; in both graphs, the observed frequency of $R_{1-2}^{N}\text{(GB)}=0$ is large, and the other states are negligible. Similarly, the comparison of the register shift distribution of Fig 5C with that of Fig 3B revealed that the two graphs are also in good agreement; only the observed frequency of $R_{2-2^{\prime }}^{N}\text{(GB)}=+1$ was large. These observations suggest that the statistical distributions of the register shifts obtained by the database analysis shown in Fig 3 are a consequence of physical interactions, rather than an evolutionary sampling bias.

### Physical explanation of the distribution of the GB loop register shift: $R_{2-2^{\prime }}^{N}\text{(GB)}\leq 0$ is prohibited by the exclusion volume condition

**Fig 6:**
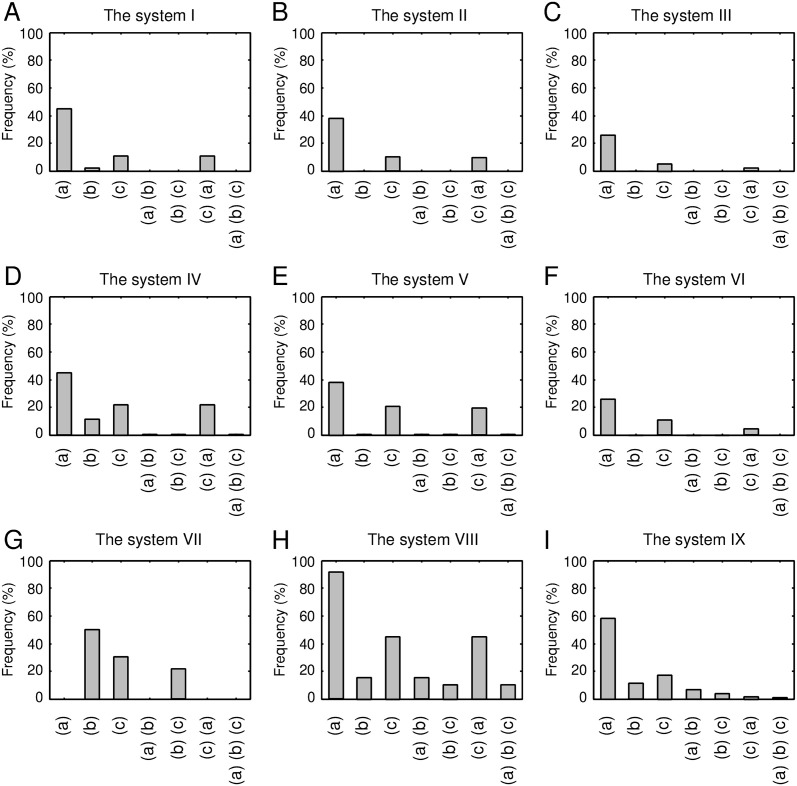
Frequency of structures that satisfied the conditions (a); (b); (c); (a) and (b); (b) and (c); (c) and (a); and all three conditions for exhaustively generated 1,586,681 structures of the systems from I to IX.

The following sections discuss the physical factors prohibiting all the systems, except for the system VIII. As data for consideration, frequencies of structures satisfying the condition (a); (b); (c); (a) and (b); (b) and (c); (c) and (a); and all three conditions for each of the systems (from I–IX) are shown in Fig 6A–6I. We excluded the system VII from the following discussion because it cannot satisfy the condition (a) at all.

First, let us discuss the systems with a register shift of $R_{2-2^{\prime }}^{N}\text{(GB)}\leq 0$, i.e., the systems I-VI. Fig 6B, 6C, 6E and 6F show that the systems II, III, V, and VI were mostly prohibited by condition (b) alone, i.e., the exclusion volume condition, as their surviving percentages were 2 × 10^−3^%, 6 × 10^−4^%, 0.5%, and 0.2%, respectively. Different from these systems, when the condition (b) alone was imposed, the surviving percentages of the systems I and IV were not nearly zero; they were 2.0% and 11%, respectively. In addition to the condition (b), if the condition (a) or (c) was added, the surviving percentages of the systems I and IV become almost zero. Note that the satisfaction of the condition (a) in these two systems corresponded to the formation of the HB3 bond (see S20 Fig). Moreover, we confirmed that all structures satisfying the condition (c) of the systems I and IV formed HB3 bond; i.e., all structures satisfying the condition (c) of the systems I and IV were included in those satisfying condition (a). These results imply that the systems I and IV cannot achieve collision-free structures while forming the hydrogen bond required for the systems.

**Fig 7:**
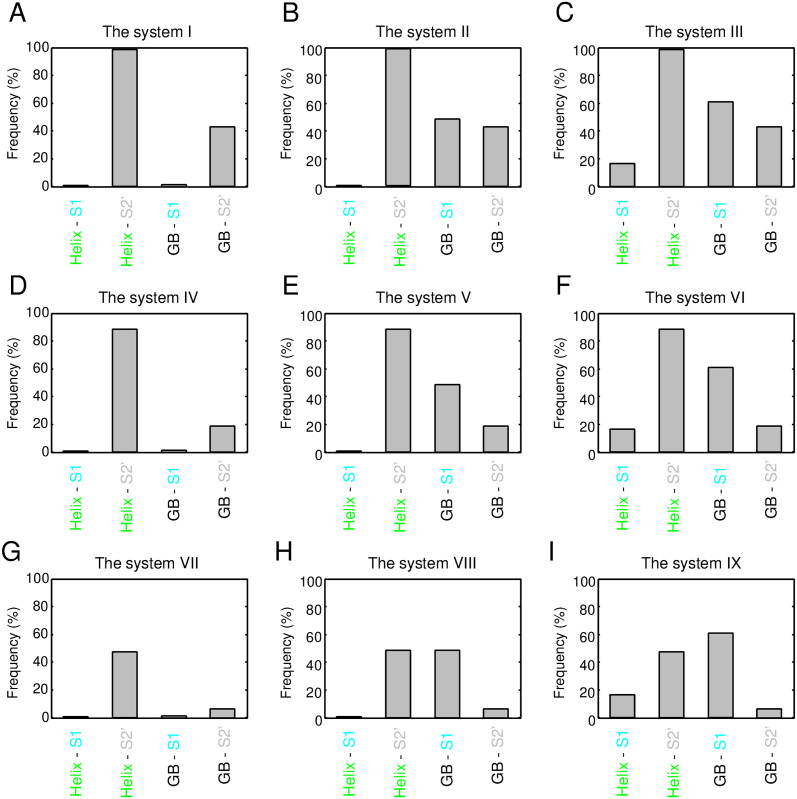
Frequency of structures with inter-atomic collisions between a given secondary structure pair for each of the system (from I to IX). The collision probability between the *α*-helix and S2 is not shown, because structures with such inter-atomic collisions were precluded before the implantation of the *αβ*-unit into the three-stranded *β*-sheet.

**Fig 8:**
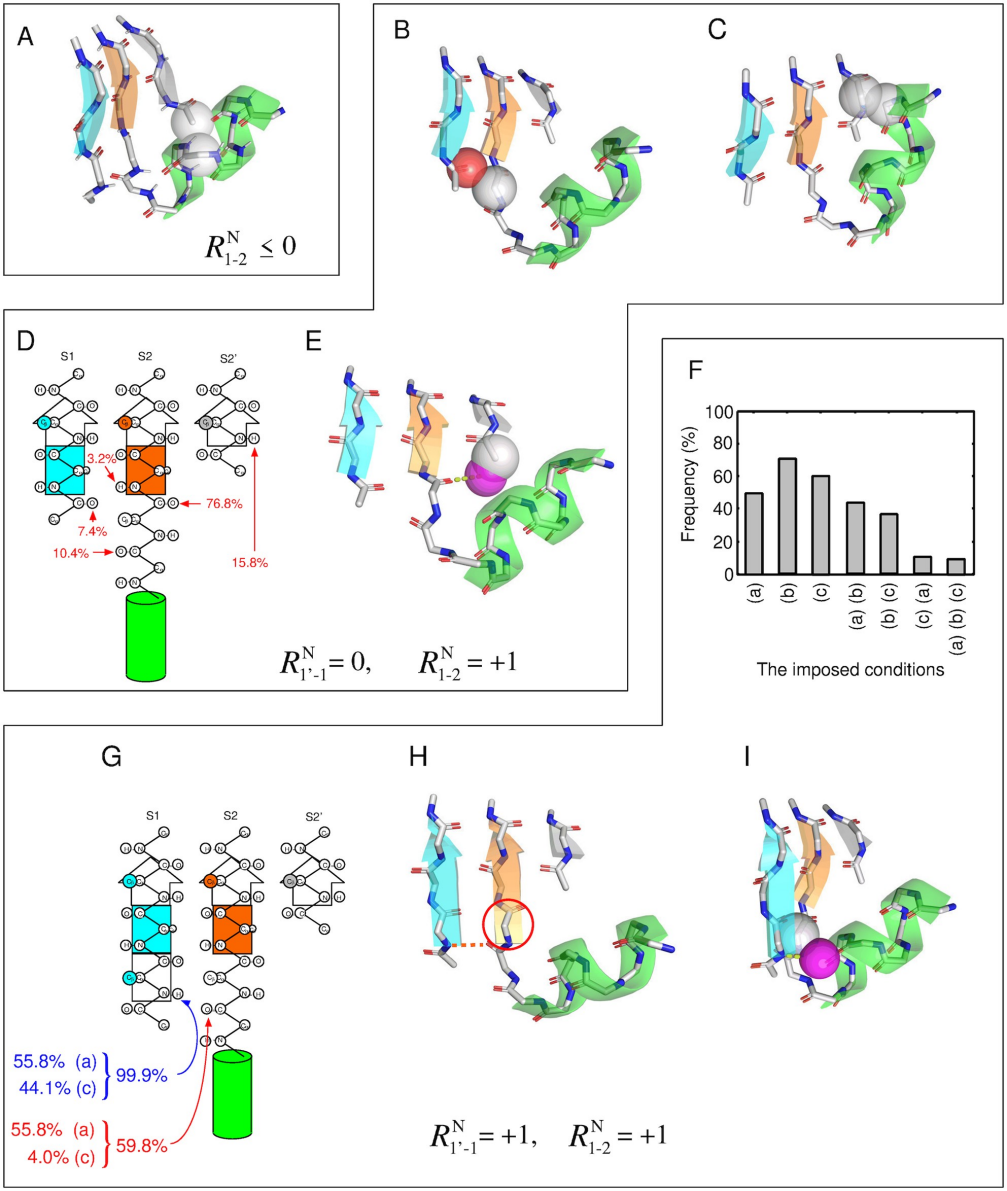
Disfavored structures of a GB loop with various three-stranded *β*-sheets identified by the simulation. The structures are categorized according to their register shifts. (A) Conformation of the system V exhibiting inter-atomic collision between the helix and S2’. This atomic collision was one of the dominant factors for the decrease of structures satisfying the condition (b) not only in the system V, but also in the systems with $R_{2-2^{\prime }}^{N}\leq 0$. (B)-(D) Disfavored conformations of the system VIII. (B) Conformation with inter-atomic collision between S1 and the GB loop and (C) that between the helix and S2’. (D) Unsatisfied polar groups and their frequencies of unsatisfaction. (E) Conformation in which the most frequently unsatisfied hydrogen bond donor did not meet the hydrogen bond satisfaction condition. (F)-(I) Disfavored conformations of the system IX. (F) Frequency of structures that satisfied the various conditions imposed on the structures corresponding to the survivors of the system VIII. (G) Polar group that violate the condition (a) or (c) and their frequencies. (H) Conformation that did not satisfy condition (a). (I) Conformation in which the most frequently unsatisfied hydrogen bond donor did not meet the condition (c). In (A), (B), (C), (E), and (I), protein atoms and virtual water molecules that collide with other atoms are depicted as transparent spheres colored in CPK and purple, respectively.

As described above, the systems with $R_{2-2^{\prime }}^{N}\text{(GB)}\leq 0$ are primarily prohibited by the exclusion volume condition. Therefore, in which atomic pairs do atomic collisions occur? Fig 7 shows the frequency of structures with inter-atomic collisions between a given secondary structure pair for each system (from I to IX). For the systems with $R_{2-2^{\prime }}^{N}\text{(GB)}=-1$ (i.e., I, II, and III), the percentage of inter-atomic collisions between the helix and S2’ was approximately 100%. Similarly, for the systems with $R_{2-2^{\prime }}^{N}\text{(GB)}=0$ (i.e., IV, V, and VI), the percentage was as high as 90%. In contrast to these systems, the systems with $R_{2-2^{\prime }}^{N}\text{(GB)}=+1$ (i.e., VII, VIII, and IX) showed the inter-atomic collisions with a probability of only about 50%. These results suggest that structures with $R_{2-2^{\prime }}^{N}\text{(GB)}\leq 0$ are almost prohibited because of an atomic crash between the helix and S2’, and that several structures without inter-atomic collisions are possible only with a register shift of $R_{2-2^{\prime }}^{N}\text{(GB)}=1$. Fig 8A shows an example of a typical structure of the system V, i.e., a structure with a register shift of $\left(R_{1-2}^{N}\text{(GB)},R_{2-2^{\prime }}^{N}\text{(GB)}\right)=\left(0,0\right)$, with the inter-atomic collision between the helix and S2’. In this figure, the atomic overlap between the C_*α*_ atom in the helix and the C_*α*_ atom in S2’ is represented as gray-colored transparent van der Waals spheres. As shown in this figure, the C-terminal part of the helix was close to S2’, and an inter-atomic collision occurred.

### Rationale for reducing the number of structures of the system VIII

The next question is why many structures in the system IX are forbidden. To address this question, we divide the structures of the system IX into structures that are prohibited for reasons common to those that are prohibited in the system VIII, and those that are prohibited for reasons specific to the system IX. This approach will facilitate the understanding of the significant difference in the number of allowed structures between the systems VIII and IX, although they differ by only one residue in the length of S1.

For this reason, we first determined the structures that are forbidden in the system VIII, and revealed the mechanism underlying this phenomenon. Fig 6H shows that the largest impact of one condition alone was condition (b), the exclusion volume condition, among the three conditions: the percentages of the surviving structures when imposing condition (a), (b), and (c) on the system VIII were about 91%, 15%, and 45%, respectively. The percentage of the surviving structures for only the condition (b) was relatively close to that observed imposing all the three conditions (10.45%), suggesting that the main cause of the reduction in the system VIII is the exclusion volume condition. Next, we discuss the percentage of the surviving structures when two conditions were imposed. The smallest percentage was observed for the combination of the condition (b) and (c) (i.e., the exclusion volume and the hydrogen bond satisfaction conditions), which was small compared with the other combinations: The percentages of the combinations of (a) and (b); (b) and (c); and (c) and (a) were 15.79%, 10.48%, and 45. 23%, respectively. The surviving percentage under conditions (b) and (c) was close to that under all the three conditions (10.45%), indicating that the combination of the conditions (b) and (c) was the dominant factor in the reduction of the system VIII.

Subsequently, we examined which secondary structure pairs frequently collide in the system VIII. Fig 7H shows that the secondary structure pairs with a high collision percentage in the system VIII were the GB loop-S1 pair and the helix-S2’ pair: Their collision percentages were 47.79% and 46.71%, respectively. The frequency of collision for either of these two secondary structure pairs was 83.1% (i.e., the frequency of the two secondary structure pairs colliding simultaneously was 11.40%), which was close to the decrease caused by inter-atomic collisions when considering all atoms in the system (see Fig 6H). This observation implies that the main factor in the decrease under the condition (b) is the collisions between these two secondary structure pairs. Fig 8B and 8C show a structure with an atomic collision between the GB loop and S1 and that with a collision between the helix and S2’, respectively.

Next, we investigated which polar groups of the system VIII fail to satisfy the condition (c), i.e., the hydrogen bond satisfaction condition. As described above, the percentage that satisfied the condition (b) alone (15.79%) was further reduced to 10.48% when the condiition (c) was added, which is nearly equal to the percentage under all the three conditions (10.45%). Here, we focused on the decrease in the percentage (5.31%). Regarding this decrease, the frequencies that each polar group failed to form a hydrogen bond are shown in Fig 8D. The largest contributor to the decrease was the CO group located just before S2, and it failed to form a hydrogen bond in 72% of the cases of the decrease. Since the other polar groups have a small percentage of unsatisfied hydrogen bonds compared with the CO group, the main contributor to the decrease was the unsatisfied hydrogen bonding of the CO group. Fig 8E shows an example of a structure with the most unsatisfied CO group not satisfying the hydrogen bond satisfaction condition. As shown in the figure, the N-terminal part of S2’ collided with the virtual water molecule generated from the CO group, implying that the CO group cannot form a hydrogen bond with a water molecule.

Taken together, these findings reveal the existence of three dominant factors for the decrease of structures satisfying the conditions in the system VIII: the atomic collisions between the GB loop and S1, those between the helix and S2’, and the unsatisfied hydrogen bond between the CO group just before S2 and a water solvent. Although the number of structures that satisfied the conditions was decreased due to the aforementioned reasons, the system VIII exhibits a survival of about 10% for the exhaustively generated structures, unlike the other systems.

To understand whether the value 10% can be considered as a relevant value or not, we compared this value with a surviving percentage of an *α*-helix formation among exhaustively generated structures. The detailed description of the *α*-helix system and the evaluating procedure are documented in Materials and methods section. The surviving percentage of the *α*-helix formation was found to be 2.3%, which was smaller than that of the system VIII, implying that the system VIII is more favorable in terms of a number of conformations that satisfied the conditions than the *α*-helix system. Thus, the value of 10% is a relevant.

### Factors that reduce the number of structures of the system IX for reasons specific to the system IX

Finally, we discuss the mechanism underlying the decrease in the number of structures of the system IX for reasons specific to the system IX. Fig 8F shows frequencies of structures that satisfied the various conditions imposed on the 165,911 structures of the system IX, which corresponded to the survivors of the system VIII. The figure demonstrates that imposing a single condition alone did not drastically reduce the number of survivors: The percentages of survivors, when conditions (a), (b), and (c) were imposed, were 49.8%, 70.3%, and 60.4%, respectively. Next, we consider the case with the two conditions imposed. The smallest percentage of survivors was observed for the combination of the conditions (c) and (a) (i.e., the hydrogen bonding satisfaction condition and the *β*-sheet hydrogen bonding), which was significantly small compared with the other combinations: the percentages of the survivors under the conditions (a) and (b); (b) and (c); and (c) and (a) were 43.5%, 36.4%, and 10.2%, respectively. The percentage of survivors under the condition (c) and (a) was close to that under the three conditions (9.6%), suggesting that the dominant factor reducing the system IX for reasons specific to the system IX is the inability to simultaneously satisfy the conditions (c) and (a).

We then identified which polar groups of the system IX cannot satisfy the two conditions. Fig 8G shows the polar groups that failed to satisfy either of the two conditions for the 165,911 structures. There were only two polar groups; the NH group located in the starting residue of S1 and the CO group in the GB loop, indicated by blue and red arrows, respectively. The NH group did not satisfy the conditions (c) or (a) in 99.9% of the decrease. On the other hand, the CO group did not satisfy the conditions in only 59.8%, implying that the dominant reason for the decrease is the inability of the NH group to satisfy the two conditions.

How does this NH group not satisfy the two conditions? As indicated in Fig 8G, this NH group did not satisfy the conditions (a) and (c) in 55.8% and in 44.1% of the decrease. Thus, the frequencies of not meeting the conditions were approximately the same for the two conditions. It is worth commenting that, when focusing only on the NH group, there was no structure that did not satisfy both conditions (a) and (c) because the unsatisfaction of the condition (a) (the HB1 bond is formed) automatically leads to the satisfaction of the condition (c) (the hydrogen bond satisfaction condition). Conversely, the unsatisfaction of the condition (c) (the NH is not hydrogen bonded) automatically leads to the satisfaction of the condition (a) (the HB1 bond is not formed). Thus, structures that satisfy the condition (a) and those that satisfy the condition (c) are exclusive for the NH group.

Fig 8H shows an example of a structure of the system IX that does not satisfy the condition (a). Recall that the satisfaction of condition (a) implies that the HB1 bond must not be formed (see S20 Fig). If the HB1 bond is formed, S2 extends one residue to the N-terminal side and the register shift becomes $R_{1-2}^{N}\text{(GB)}=0$, as shown in Fig 8H, which is a different register shift required for the system IX. In addition, the formation of the HB1 bond converts the GB loop into the G loop. Therefore, the tendency to form the HB1 bond is a significant factor that hampers generating structures of the system IX. The next example is a structure that does not satisfy condition (c) (see Fig 8I). The figure indicates that the virtual water molecule generated from the NH group collides with C_*α*_ atoms in the GB loop. Since the NH group and the GB loop are close in space, the NH group cannot form hydrogen bonds with a water molecule. Taken together, these findings suggest that the fact that system IX has the NH group explains the large discrepancy in the number of allowed structures observed between the system VIII and IX, as it has no choice but to form intramolecular HB1 bond or stay in a state of hydrogen bond unsatisfaction.

### Lessons from *de novo* designed proteins with blueprints that violate the register shift rules

**Fig 9:**
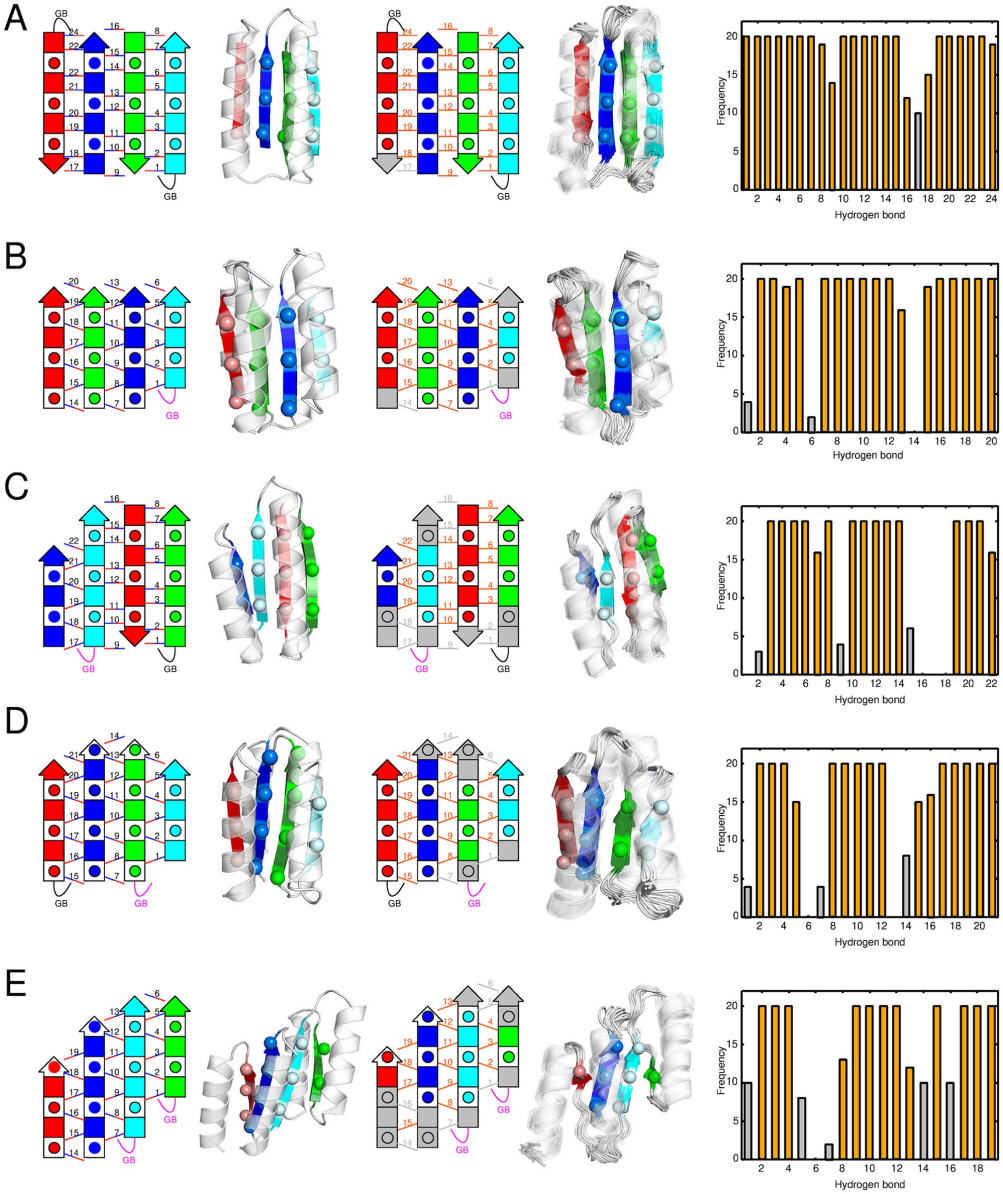
The degree of agreement between the blueprints and the NMR structures for the five *de novo*-designed proteins reported in Ref. [3]. Left to right: the blueprints, the target structures, the blueprints colored according to their consistency with their NMR structures, the NMR structures, and frequency of the hydrogen bonds formation in the NMR structures. On the rightmost column, hydrogen bonds with a forming probability of 50% or less are indicated by gray bars, and the others are indicated by orange bars. Correspondingly, on the third column from the left, hydrogen bonds with a forming probability of 50% or less are indicated by gray lines, the other hydrogen bonds are indicated by orange lines, and residues that were designated as *β*-strands in the blueprint, but form a *β*-strands with a probability of 50% or less are colored in gray. (A) Fold-I, (B) Fold-II, (C) Fold-III, (D) Fold-IV, and (E) Fold-V.

From the above arguments, we have confirmed that the register shift rule for a GB loop deduced from the database analysis is a consequence of physical interactions. The rule for a GB loop states that $R_{1-2}^{N}\text{(GB)}$ must be zero, and $R_{2-2^{\prime }}^{N}\text{(GB)}$ must be greater than or equal to 1. Here, we examine the consequences of performing *de novo* protein design based on a blueprint that violates the register shift rules for GB loops. We found that, in the blueprints of the five *αβ* fold proteins designed in Ref. [3], some GB loops violated the rules. Let us compare these blueprints with their corresponding structures determined by nuclear magnetic resonance (NMR). Fig 9 shows the blueprints used in Ref. [3](the first column), the three-dimensional target structures generated based on the blueprints (the second column), the blueprints colored according to the consistency with their NMR structures (the third column), the NMR structures (the fourth column), and the frequency of hydrogen bond formation calculated based on the NMR structures (the fifth column). In the first and third columns of the figure, the black curves, the magenta curves, and the gray-colored filled rectangles represent GB loops that satisfy the rule; those that violate the rule; and residues that were designated to *β*-strands in the blueprints, but did not form in the NMR structures; respectively. Although the NMR structures were consistent with the target structures in terms of RMSD, they were not consistent with the hydrogen bonds specified in the blueprints. Note that all hydrogen bonds located near the GB loops that violated the rules were not formed in the NMR structure. In contrast, those that satisfied the rules, with one exception, were well-formed, and the exception can be rationalized: the one exception was the hydrogen bond 1 in Fold III (Fig 9C), which was disrupted by the lack of formation of *β*-strand of the C-terminal residue of the red-colored *β*-strand; This phenomenon is a consequence of violating the $R_{2-2^{\prime }}^{N}\text{(GB)}\geq 1$ rule. Additionally, the phenomenon that N-terminal residues of the red- and blue-colored *β*-strands were not formed in the NMR structure of Fold V (Fig 9E) can be interpreted as the domino effect of breaking *β*-strand residues initiated by violating the $R_{1-2}^{N}\text{(GB)}=0$ rule of a GB loop that is connected to the cyan-colored *β*-strand. Such failures, when they occur in proteins consisting of a larger number of *β*-strands, could lead to more serious design failures. For more a complete and accurate *de novo* design of *αβ*-proteins, the register shift rules are necessary and will play an important role in creating appropriate blueprints.

## Materials and methods

### Mathematical definition of the register shift

This section describes the mathematical definition of the register shifts. We consider four-stranded *β*-sheets consisting of the S1’, S1, S2, and S2’ *β*-strand, as shown in Fig 1D. For these *β*-sheets, the register shifts $R_{1^{\prime }-1}^{N}$, $R_{1-2}^{N}$, $R_{2-2^{\prime }}^{N}$, $R_{1^{\prime }-1}^{C}$, $R_{1-2}^{C}$, and $R_{2-2^{\prime }}^{C}$ are defined as follows.

$$\begin{array}{ccc} R_{1^{\prime }-1}^{N} & = & \left\{ \begin{array}{cc} |i^{N}\left(\text{S1}’,\;\text{S1}\right)-i^{N}\left(\text{S1}’\right)|-|i^{N}\left(\text{S1},\;\text{S1}’\right)-i^{N}\left(\text{S1}\right)| & \left(\text{parallel}\right)\\ |i^{C}\left(\text{S1}’,\;\text{S1}\right)-i^{C}\left(\text{S1}’\right)|-|i^{N}\left(\text{S1},\;\text{S1}’\right)-i^{N}\left(\text{S1}\right)| & \left(\text{anti}\;\text{parallel}\right) \end{array}\right.\\ R_{1-2}^{N} & = & |i^{N}\left(\text{S1},\;\text{S2}\right)-i^{N}\left(\text{S1}\right)|-|i^{N}\left(\text{S2},\;\text{S1}\right)-i^{N}\left(\text{S2}\right)|\\ R_{2-2^{\prime }}^{N} & = & \left\{ \begin{array}{cc} |i^{N}\left(\text{S2},\;\text{S2}’\right)-i^{N}\left(\text{S2}\right)|-|i^{N}\left(\text{S2}’,\;\text{S2}\right)-i^{N}\left(\text{S2}\right)| & \left(\text{parallel}\right)\\ |i^{N}\left(\text{S2},\;\text{S2}’\right)-i^{N}\left(\text{S2}\right)|-|i^{C}\left(\text{S2}’,\;\text{S2}\right)-i^{C}\left(\text{S2}\right)| & \left(\text{anti}\;\text{parallel}\right) \end{array}\right.\\ R_{1^{\prime }-1}^{C} & = & \left\{ \begin{array}{cc} |i^{C}\left(S1,S1^{\prime }\right)-i^{C}\left(S1\right)\left|-\right|i^{C}\left(S1^{\prime },S1\right)-i^{C}\left(S1^{\prime }\right)| & \left(\text{parallel}\right)\\ |i^{C}\left(S1,S1^{\prime }\right)-i^{C}\left(S1\right)\left|-\right|i^{N}\left(S1^{\prime },S1\right)-i^{C}\left(S1^{\prime }\right)| & \left(\text{anti}\;\text{parallel}\right) \end{array}\right.\\ R_{1-2}^{C} & = & |i^{C}\left(S2,S1\right)-i^{C}\left(S2\right)|-|i^{C}\left(S1,S2\right)-i^{C}\left(S1\right)|\\ R_{2-2^{\prime }}^{C} & = & \left\{ \begin{array}{cc} |i^{C}\left(S2^{\prime },S2\right)-i^{C}\left(S2^{\prime }\right)\left|-\right|i^{C}\left(S2,S2^{\prime }\right)-i^{C}\left(S2\right)| & \left(\text{parallel}\right)\\ |i^{N}\left(S2^{\prime },S2\right)-i^{N}\left(S2^{\prime }\right)\left|-\right|i^{C}\left(S2,S2^{\prime }\right)-i^{C}\left(S2\right)| & (\text{anti}\;\text{parallel}). \end{array}\right. \end{array}$$

Here, *i*^*N*^(*X*) and *i*^*C*^(*X*) are the residue number of the N-terminal and C-terminal residues of *β*-strand *X*, respectively. *i*^*N*^(*X*, *Y*) and *i*^*C*^(*X*, *Y*) are the most N-terminal and the most C-terminal residue among residues of strand X forming a cross-strand residue pairing with a residue of strand Y, respectively. An explanation of these variables in graphical form is given in S1 Fig.

### The reason for the choice of the five-residue *β*-strand for the consensus three-stranded *β*-sheet structure

We used the five-residue *β*-strands for the consensus three-stranded *β*-sheet structure. The reasons are as follows: First, as shown in S11–S19 Figs, atoms with over five residues are required for the sysmtes. Second, by choosing a five-residue length *β*-strand, the effect of the loop structure attached to the *β*-strand can be eliminated almost totally. As shown in S11–S19 Figs, not all five residues need to be *β*-strand; the middle three residues of the five residue *β*-stranded structure are sufficient. However, if the edge of a five-residue peptide is a loop, the structure of the edge residue and that of the residues spatially adjacent to it can be optimized for its specific loop structure. To eliminate this dependency on the loop structure, we chose the five-residue *β*-strands for the consensus *β*-sheet.

### The procedure for implanting the structure of the *αβ*-unit into the three-stranded *β*-sheet structure

This section provides the procedure for implanting the structure of the *αβ*-unit into the three-stranded *β*-sheet structure. A schematic representation of the procedure is presented in S21 Fig. The C and O atoms of the 11th residue and the N, CA, C, and O atoms of the 12th residue of the *αβ*-unit structure are superimposed on the C and O at the 108th residue and the N, CA, C, and O atoms of the 109th residue of the consensus *β*-sheet structure, as shown in S10 Fig, respectively, so that their RMSD was minimized. To remove the redundant atoms, the atoms of the S2 *β*-strand of the consensus *β*-sheet used for the superposition were removed from the system.

### The procedure for calculating surviving percentage of an *α*-helix system

We used an 11-residue peptide as a model system for an *α*-helix (see S22A Fig). For this system, we selected an amino acid sequence made solely of alanine residues. All bond lengths and bond angles were fixed to the standard values defined in the CHARMM Param 19 parameter set [29]. All *ϕ*, *ψ* and *ω* angles were also fixed to the typical values of *α*-helix ((*ϕ*, *ψ*, *ω*) = (−60, −45, 180)), except for *ϕ* and *ψ* angles of the central three residues of the model peptide (5th, 6th and 7th residue). The *ϕ* and *ψ* angles of the central three residues of the model peptide were exhaustively sampled within the popular region of the A region of the ABEGO classification (S22B and S22C Fig). The threshold for the popular regions was determined so that the total number of sampling structures were roughly equivalent to the number of structure exhaustively generated in the simulation of the system VIII (1,586,681). The resultant popular A region had 117 discrete states, thus, the total number of exhaustively sampled structures was = 117^3^ = 1, 601, 613. The surviving percentage was calculated by counting the number of structures that formed all the hydrogen bonds required for an *α*-helix.

## Supporting information

S1 Fig A graphical explanation of *i*^*N*^(*X*), *i*^*C*^(*X*), *i*^*N*^(*X*, *Y*), and *i*^*C*^(*X*, *Y*), *i*^*N*^(*X*) and *i*^*C*^(*X*) are the residue number of the N-terminal and the C-terminal residue of the X *β*-strand. *i*^*N*^(*X*, *Y*) and *i*^*C*^(*X*, *Y*) are the most N-terminal and the most C-terminal residue among residues of the X *β* strand that form a cross-strand residue pairing with a residue of Y strand. The gray arrows indicate residue pairs that form a cross-strand residue pairing. *i*^*N*^(*X*), *i*^*N*^(*X*, *Y*), *i*^*C*^(*X*, *Y*), *i*^*C*^(*X*), *i*^*N*^(*Y*), *i*^*N*^(*Y*, *X*), *i*^*C*^(*Y*, *X*), and *i*^*C*^(*Y*) are colored in blue, cyan, light green, dark green, yellow, orange, red, and magenta, respectively. If the N-/C-terminal residue of the *β*-sheet is identical to the most N-/C-terminal residue among residues of the strand forming a cross-strand residue pairing, it is painted with stripes. (A) The case where the X *β*-strand and the Y *β*-strand are parallel. (B) The case where the X *β*-strand and the Y *β*-strand are anti-parallel.
(EPS)

S2 FigImpact of the location of *βαβ*-motifs at the end of the *β*-sheet on the possible values of the register shift.(A) Schematic representation of the *β*-sheet structure with a register shift of $R_{1-2}^{N}=+1$ (left) and $R_{1-2}^{C}=-1$ (right) in the case of S1 being located at the edge of the *β*-sheet. These two structures are prohibited because there is no hydrogen bonding partner for the residues located at the end of S1. (B) Schematic representation of the *β*-sheet structure with a register shift of $R_{1-2}^{N}=+1$ (left) and $R_{1-2}^{C}=-1$ (right) in the case of S1 not being located at the edge of the *β*-sheet. These two structures are possible, even though the register shift values are the same as in (A). (C) Schematic representation of the *β*-sheet structure with a register shift of $R_{1-2}^{N}=-1$ (left) and $R_{1-2}^{C}=+1$ (right) in the case of S2 being located at the edge of the *β*-sheet. These two structures are prohibited because there is no hydrogen bonding partner for the residues located at the end of S2. (D) Schematic representation of the *β*-sheet structure with a register shift of $R_{1-2}^{N}=-1$(left) and $R_{1-2}^{C}=+1$(right) in the case of S2 not being located at the edge of the *β*-sheet. These two structures are possible, even though the register shift values are the same as in (C).(EPS)

S3 FigThe observed frequency of the register shift of $R_{1^{\prime }-1}^{N}$, $R_{1-2}^{N}$, and $R_{2-2^{\prime }}^{N}$, for the most frequent loop in *αβ*-units to the fifth most frequent loop.The horizontal axis is the value of register shifts. The vertical axis is the observed frequencies of the register shifts expressed as percentage.(EPS)

S4 FigThe observed frequency of the register shift of $R_{1^{\prime }-1}^{N}$, $R_{1-2}^{N}$, and $R_{2-2^{\prime }}^{N}$, for the sixth most frequent loop in *αβ*-units to the tenth most frequent loop.The horizontal axis is the value of register shifts. The vertical axis is the observed frequencies of the register shifts expressed as percentage.(EPS)

S5 FigThe observed frequency of the register shift of $R_{1^{\prime }-1}^{C}$, $R_{1-2}^{C}$, and $R_{2-2^{\prime }}^{C}$, for the most frequent loop in *βα*-units to the fifth most frequent loop.The horizontal axis is the value of register shifts. The vertical axis is the observed frequencies of the register shifts expressed as percentage.(EPS)

S6 FigThe observed frequency of the register shift of $R_{1^{\prime }-1}^{C}$, $R_{1-2}^{C}$, and $R_{2-2^{\prime }}^{C}$, for the sixth most frequent loop in *βα*-units to the tenth most frequent loop.The horizontal axis is the value of register shifts. The vertical axis is the observed frequencies of the register shifts expressed as percentage.(EPS)

S7 Fig The model system of an *αβ*-unit connected by GB loops. This system consists of 12-amino acid residues. The amino acid sequence of the system is all alanine, with exception of the ninth residue, which is glycine. The secondary structure of this peptide is restricted to the *α*-helix for the first eight residues, the GB loop for the next two residues, and the *β*-strand for the last two residues. The dihedral angle of each amino acid residue is constrained to that of the ABEGO class corresponding to the respective secondary structure (depicted on the third row of the figure). The three residues in gray are subjected to an exhaustive dihedral angle search within the specified the ABEGO class. The internal coordinates of the other residues are constrained to those of the consensus structure.
(EPS)

S8 Fig Frequency of the occurrence of amino acids at the position of G in the GB loop of the *αβ*-unit in our dataset. The horizontal and vertical axis respectively represent 20 amino acid types and their observed frequency in the first position of GB loop.
(EPS)

S9 Fig Ramachandran plots obtained from the protein structure database (left) and the regions used for sampling (right). In both plots, regions are grouped into 5° × 5° bins. In Ramachandran plots, the color represents the observed frequency of a given (*ϕ*, *ψ*) angle. In the graph shown on the right, the red-colored regions represent the regions used for sampling. We chose the popular regions of the Ramachandran plot as sampling regions. Here, the threshold for the popular regions was set to 0.3%. (A) Ramachandran plots of region B. (B) The B region used for sampling. This region had 277 discrete states. (C) Ramachandran plots of region G. (D) The G region used for sampling. This region had 145 discrete states.
(EPS)

S10 Fig Atoms used in computation extracted from the three-stranded consensus *β*-sheet structure. The consensus structure is three *β*-strands extracted from the structure of the bacterial cell division regulator protein MipZ (PDB ID: 2xj4), and residues of the three-strands are 5–9, 36–40, and 106–110. We used only the heavy atoms of the main chain and CB atoms for the calculations. There are additional atoms at both the N- and C-termini of the three-residue long strands. They are atoms whose atomic coordinates are automatically determined by imposing the two conditions: dihedral angle *ω* of 180° and hydrogen bonds required to exist as a three-residue long *β*-sheet.
(EPS)

S11 Fig All atoms of the *β*-sheet of the system I. (EPS)

S12 Fig All atoms of the *β*-sheet of the system II. (EPS)

S13 Fig All atoms of the *β*-sheet of the system III. (EPS)

S14 Fig All atoms of the *β*-sheet of the system IV. (EPS)

S15 Fig All atoms of the *β*-sheet of the system V. (EPS)

S16 Fig All atoms of the *β*-sheet of the system VI. (EPS)

S17 Fig All atoms of the *β*-sheet of the system VII. (EPS)

S18 Fig All atoms of the *β*-sheet of the system VIII. (EPS)

S19 Fig All atoms of the *β*-sheet of the system IX. (EPS)

S20 Fig Definition of the intra-chain hydrogen bonds used for evaluating *β*-sheet formation. The intra-chain hydrogen bonds, HB1, HB2, and HB3, are defined as depicted in this figure. The list of the hydrogen bonds required for the system I–IX is shown in Table 1.
(EPS)

S21 Fig Schematic image of the procedure for implanting the *αβ*-unit connected by GB loops into the three-stranded *β*-sheet. (A) An example of structure of the *αβ*-unit. (B) The consensus structure of a three-strand *β*-sheet (C) An example of structure for evaluation. The cyan colored atoms of (A) are superimposed to cyan colored atoms of (B) by minimizing their RMSD.
(EPS)

S22 Fig The model system of an *α*-helix and the region of dihedral angles to be sampled. (A) The model system of an *α*-helix. The system consists of 11-amino acid residues. The amino acid sequence of the system is all alanine. The secondary structure of this peptide is restricted to an *α*-helix for all the residues. The three residues in gray are subjected to an exhaustive sampling of dihedral angles within the “A” region of the ABEGO classification. The dihedral angles of the other residues were fixed to the typical values of an *α*-helix ((*ϕ*, *ψ*) = (−60, −45)). (B) Ramachandran plot of “A” region obtained from the protein structure database and (C) the popular region of the Ramachandran plot. In the Ramachandran plot, the color represents the observed frequency of a given (*ϕ*, *ψ*) angle. In the graph shown in (C), the red-colored region represent the popular region. Here, the threshold for the popular regions was set to 0.235%. The resultant popular region had 117 discrete states. We used the popular region as the sampling region.
(EPS)
